# Co-Entrapment of Sorafenib and Cisplatin Drugs and iRGD Tumour Homing Peptide by Poly[ε-caprolactone-co-(12-hydroxystearate)] Copolymer

**DOI:** 10.3390/biomedicines10010043

**Published:** 2021-12-26

**Authors:** Izolda Kántor, Diana Dreavă, Anamaria Todea, Francisc Péter, Zoltán May, Emese Biró, György Babos, Tivadar Feczkó

**Affiliations:** 1 Institute of Materials and Environmental Chemistry, Research Centre for Natural Sciences, Magyar Tudósok Körútja 2, H-1117 Budapest, Hungary; kantor.izolda@ttk.hu (I.K.); may.zoltan@ttk.hu (Z.M.); emese.biro@gmail.com (E.B.); babos@mukki.richem.hu (G.B.); 2 Research Institute of Biomolecular and Chemical Engineering, Faculty of Engineering, University of Pannonia, Egyetem u. 10, H-8200 Veszprém, Hungary; 3 Faculty of Industrial Chemistry and Environmental Engineering, University Politehnica Timişoara, Carol Telbisz 6, RO-300001 Timişoara, Romania; diana.dreava@upt.ro (D.D.); francisc.peter@upt.ro (F.P.); 4 Research Institute for Renewable Energies, University Politehnica Timișoara, G. Muzicescu 138, RO-300501 Timișoara, Romania

**Keywords:** polymeric nanoparticles, drug encapsulation, biobased oligomers, sorafenib, cisplatin

## Abstract

The drug-loaded nanocarriers have overcome various challenges compared with the pure chemotherapeutic drug, such as limited bioavailability, multiple drug resistance, poor patient compliance, and adverse drug reactions, offering advantages such as protection from degradation in the blood stream, better drug solubility, and improved drug stability. One promising group of controlled and targeted drug delivery systems is polymer-based nanoparticles that can sustain the release of the active agent by diffusion and their degradation. Sorafenib is the only drug that can prolong the life of patients suffering from hepatocellular carcinoma. Cisplatin remains one of the most widely used broad-spectrum anticancer drugs for the treatment of a variety of solid tumours. Nanoformulations can exert a synergistic effect by entrapping two drugs with different modes of action, such as sorafenib and cisplatin. In our study, polymeric nanoparticles were prepared with a good production yield by an improved double emulsion solvent evaporation method using the copolymer of 12-hydroxystearic acid with ε-caprolactone (12CL), a biocatalytically synthesised biocompatible and biodegradable carrier, for the co-entrapment of sorafenib and cisplatin in nanotherapeutics. A bovine serum albumin (BSA) model compound was used to increase the cisplatin incorporation; then, it was successfully substituted by a iRGD tumour penetrating peptide that might provide a targeting function of the nanoparticles.

## 1. Introduction

Nanomedicine is one of the most rapidly developing branches of pharmaceutics and can be defined as nanotechnology that uses materials with nanoscale size, which are applied to health and medicine [1]. Current problems in cancer treatment include low specificity, rapid drug clearance and biodegradation, and limited targeting [2]. The characteristics of nanocarriers, such as size, high surface-to-volume ratios, drug release profiles, and the ability to modify them for targeting, enable them to penetrate efficiently into the targeted tumour tissue and release drugs in a stable and controlled way [3]. Nanocarriers have a wide spectrum of composition and various morphologies; furthermore, their chemistry was modified to improve loading efficiency and delivery capacity [4].

In the list of most outstanding developments, there is a broad range of engineered and functionalised nanoparticles for site-specific targeting of therapeutic agents, such as sorafenib and cisplatin, to the cancer area [5]. Significant outcomes have been recorded in clinical investigations, especially for some types of cancer and microbial infection treatments [6]. Currently, there are several clinically approved nanoscale materials that are promising nanoparticle drug delivery systems that have shown reduced side effects and effective therapeutic levels [7].

Liver cancer is the sixth most common cancer in the world, and its mortality rate ranks second among all cancers. The most common type of liver cancer is hepatocellular carcinoma [8]. Patients in the intermediate and advanced stages may be considered for chemoembolisation and sorafenib therapies. Treatment with sorafenib, a member of the class of phenylureas, is known to increase the survival rate of patients suffered from hepatocellular carcinoma [9]. Sorafenib is an orally active multikinase inhibitor drug. In addition, sorafenib is used for the treatment of kidney cancer and thyroid cancer [10]. The most common drug-related side effects are diarrhoea and hand–foot skin reaction [11].

Cisplatin, *cis*-diamminedichloroplatinum(II), is one of the most widely used anticancer drugs. It has a broad spectrum for the treatment of many tumours, including sarcomas, carcinomas, liver, lung, ovarian, and cervical cancer [12]. Cisplatin is administered intravenously and interferes with DNA replication, killing cancer cells [13]. Its use is associated with serious side effects, such as kidney and hearing problems, and nerve damage [14].

Some of the disadvantages of these anticancer active agents are that they are quickly recognised and removed by the immune system of the body before they reach their target. Since they are not targeted in the body during their administration, their dose must be increased to reach the therapeutic level causing serious side effects. Nanoscale drug carriers offer many advantages compared to pure chemotherapeutics, such as protection against degradation in the blood stream, better drug solubility, and improved drug stability [15].

With controlled and targeted drug delivery, drug toxicity, adverse effects, and administered doses can be reduced; furthermore, these systems can deliver drugs over an extended period or a specific treatment period [16]. Therefore, the improvement of controlled and targeted drug delivery systems became essential, especially in the past two decades, when many nanoparticle systems have been developed for efficient and controlled drug delivery [17]. One type of the controlled and targeted drug delivery systems consists of polymer-based nanoparticles, which can sustain the release of the active agent by diffusion and their degradation. Polymer nanoparticles show better stability, more homogeneous particle size, improved drug circulation time, and more controlled drug delivery compared to micelles and liposomes [18].

The encapsulation of proteins into nanoparticles is widely used nowadays because their release kinetics can be controlled, the secondary structure of proteins is preserved and, in addition, significant amounts of drug can be incorporated into the particle matrix due to the large number of drug binding sites on the protein molecule [19]. Bovine serum albumin is extensively used as a material for drug delivery, showing several advantages, such as biodegradability, non-toxicity, and non-immunogenicity, as well as a dual targeting ability to tumour tissues [20]. An important active targeting agent is the iRGD tumour penetration peptide, known as the tumour homing peptide [21]. iRGD preferentially binds to integrin receptors which are overexpressed by cancer cells. After binding, the peptide activates a pathway through cancer cells [22]. This pathway can be used as an improved delivery route for the anticancer drugs [23].

The aim of this work was to prepare anticancer drug-loaded polymeric nanoparticles from a biodegradable and biocompatible oligomer, co-encapsulating sorafenib and cisplatin, which can become effective sustained and targeted drug delivery devices. The oligomer was obtained through a green biocatalytic route, from ε-caprolactone and 12-hydroxystearic acid using a microbial lipase, as previously reported [24], and was proved to be efficient for the nanoencapsulation of sorafenib [25]. It belongs to a new class of biobased oligomers of hydroxy acids, which are increasingly considered for biomedical and cosmetic applications [26]. Polyesters synthesised by enzymatic catalysis attracted special interest in the last period as drug delivery platforms, particularly for anticancer treatments [27]. Most of them are in fact oligoesters, due to the limitations of in vitro biocatalysis in achieving high molecular weights, but this characteristic represents an advantage, allowing better solubility in the organic phase during the encapsulation by the double emulsion evaporation method, as well as considering its better biodegradability. Nevertheless, the simultaneous encapsulation of two active drugs represents a difficult task, particularly when cisplatin is targeted. The double emulsion solvent evaporation method was optimised for the preparation of nanoparticles. Particle size distribution, production yield, and encapsulation efficiency of the active agents were investigated. In vitro drug release and in vitro cytotoxicity tests were also carried out. In preliminary experiments, BSA was used as an amino acid-based additive to protect and enhance the incorporation of cisplatin. Subsequently, BSA was replaced by the iRGD tumour penetration peptide, which is an active targeting agent for cancer cells. Co-encapsulation of sorafenib and cisplatin was achieved for the first time in the present work and could represent a relatively easy practical way for the targeted delivery of more than one active drug.

## 2. Materials and Methods

### 2.1. Materials

ε-Caprolactone-12-hydroxystearic acid copolymer (12CL) polyester was synthesised biocatalytically in our previous work [25]. Polyvinyl alcohol (PVA, Mw = 30,000–70,000 g/mol, 87–90% hydrolysed), dichloromethane (DCM), 1-ethyl-3(3-dimethylaminopropyl) carbodiimide (EDC), N-hydroxy succinimide (NHS), N,N-dicyclohexylcarbodiimide (DCC), acetone, dimethyl sulfoxide (DMSO), sodium azide, sodium acetate, iRGD, sodium dodecyl sulfate (SDS), 3-(4,5-dimethylthiazol-2-yl)-2,5-diphenyltetrazolium bromide (MTT) and Dulbecco’s Modified Eagle’s medium (DMEM (high glucose)), 12-hydroxystearic acid (98%), ε-caprolactone, trans-2-[3-(4-t-butyl-phenyl)-2-methyl-2-propenylidene]malononitrile (DCTB), and potassium trifluoroacetate (KTFA), were obtained from Sigma–Aldrich (St. Louis, MO, USA). Sorafenib (free base) was purchased from Active Biochem (Hong Kong, China), cis-dichlorodiamineplatinum(II) and 99.99% (trace metal basis) was from Acros Organics (Geel, Belgium). Micro bicinchonionic acid (micro BCA) protein assay kit and bovine albumin (fraction V) (BSA) were bought from the Thermo Fisher Scientific (Waltham, MA, USA), iRGD peptide from GenScript USA Inc. (Piscataway, USA), and nitric acid 68–70% (HNO3) from Scharlab (Barcelona, Spain). Hydrochloric acid (HCl) 37% and glacial acetic acid were products of VWR International S.A.S (Rosny-sous-Bois, France). The HepG2 human hepatic carcinoma cell line was a kind gift from the National Institute of Oncology (Budapest, Hungary). The Novozym 435 lipase was from Novozymes (Bagsvaerd, Denmark).

### 2.2. Biocatalytic Synthesis and Characterisation of the ε-Caprolactone-12-hydroxystearic Acid Copolymer

The synthesis protocol reported previously [24] was slightly modified and scaled-up at gram level. ε-Caprolactone (570 mg, 5 mmole) and 12-hydroxystearic acid (300 mg, 1 mmole), at 5:1 molar ratio, were added to 10 mL toluene in a 25-mL round bottom flask. The mixture was heated at 80 °C and stirred until a homogeneous mixture was obtained. Then, 50 mg lipase from *Candida antarctica* B immobilised on acrylic resin (commercial name Novozym 435) were added, the flask was attached to a rotary evaporator (Heidolph Laborota 4000 efficient, Heidolph Instruments, Schwabach, Germany) and the reaction was run under gentle stirring (60 rpm) at the same temperature for 48 h, without applying vacuum. At the end of the reaction, the enzyme was removed by filtration, and then the solvent was evaporated. The polymerisation product was analysed by MALDI-TOF MS, without additional purification steps.

MALDI-TOF MS assay was carried out using an UltrafleXextreme Bruker spectrometer with FlexControl and FlexAnalysis software packages for acquisition and processing of the data (BrukerDaltonics, Germany), at an acceleration voltage of 25 kV. DCTB has been used as a matrix and KTFA as ionisation agent, following the already described steps of sample preparation and analysis [28]. Estimation of the number average molecular weight and composition of the oligomerisation product by MALDI-TOF MS (the spectrum is presented in Figure S1, Supplementary Material) gave 1450 Da and 75% linear copolymer content, respectively.

### 2.3. Preparation of Nanoparticles by Double Emulsion Solvent Evaporation Method

The dual drug-loaded nanoparticles were prepared by the double emulsion solvent evaporation method. Basically, the organic phase was composed of 10 mg polymer (12CL) dissolved in 1 mL DCM. In the encapsulation experiments with active agents, various amounts of sorafenib (0.5–1.0 mg) dissolved in acetone were added into the organic phase, as well as DCC cross-linking agent (1–4 mg) in the specified experiments. The inner water phase contained 0.5–1 mg of BSA or iRGD, and cisplatin. In some experiments, specified in the discussion part, EDC and NHS (each 1 mg) were also added into the inner water phase.

In the first step, the inner water phase was poured to the organic phase, and the two phases were emulsified by sonication using a Hielscher Ultrasonic Processor UP200St (Hielscher Ultrasonics GmbH, Teltow, Germany) at an amplitude of 30% for 30 s. Then, this first emulsion was transferred to the outer water phase, which was a 5 mL aqueous solution of PVA (1% (*w/v*)), and homogenised again with sonication at an amplitude of 50% for 60 s. The organic solvents were evaporated by magnetical stirring for 2 h at room temperature and under atmospheric pressure. The nanoparticles were centrifuged by a Hermle Z216 MK microcentrifuge (Hermle Labortechnik, Wehingen, Germany) with 21,380 g for 30 min, washed three times and redispersed in MilliQ water.

### 2.4. Characterisation of Nanoparticles

#### 2.4.1. Particle Size, Morphology and Zeta Potential Analysis

The size of the nanoparticles was measured by the dynamic light scattering method, using a Zetasizer Nano ZS (Malvern Instruments Ltd., Malvern, UK) instrument. The particles were characterised by their intensity mean diameter (Z-avg) and polydispersity index (PDI). The zeta potential was measured using the Laser Doppler Electrophoresis technique by the same instrument.

The nanoparticle morphology was investigated using a FEI Talos F200XG2 scanning/transmission electron microscope (S/TEM, Thermo Fischer Scientific, Waltham, MA, USA) operated at 200 keV.

#### 2.4.2. Yield and Encapsulation Efficiency

The production yield of the nanoparticles was determined by gravimetric measurement after washing and drying to a constant mass of 0.5 mL of nanoparticle suspension. The encapsulation efficiency of sorafenib was investigated by dissolving the pellet resulting from centrifugation of 0.5 mL of nanoparticle suspension in 1 mL DMSO, and the solution was diluted to be detectable in the linear calibration range (1.25–25.0 μg/mL). The absorbance of sorafenib solutions was measured by ATI Unicam UV4-100 spectrophotometer (Unicam Instruments Ltd., Cambridge, UK) at the maximum absorbance (271 nm).

The encapsulation efficiency of the cisplatin and iRGD peptide was determined by measuring the platinum and sulphur content of the nanoparticles by atomic emission spectroscopy using a Spectro Genesis ICP-OES (SPECTRO Analytical Instruments GmbH, Kleve, Germany) simultaneous spectrometer with axial plasma observation. Multielemental standard solutions were used for calibration (Teknolab Sorbent AB., Kungsbacka, Sweden). The nanoparticles were digested with 2 mL of 68–70% HNO_3_ and 1 mL of 37% HCl. After digestion, the samples were diluted with MilliQ water to reach the desired calibration range.

The encapsulation efficiency (EE) of the active agents was calculated as follows:

Encapsulation efficiency (%) = (mass of drug in nanoparticles/mass of total drug) × 100.

#### 2.4.3. Quantification of BSA by Micro Bicinchoninic Acid Protein Assay

The amount of encapsulated BSA was measured by using the Thermo Scientific^TM^ Micro BCA Protein Assay Kit (Product No. 23225). After the washing process, 0.5 mL of supernatant was taken, and the same amount of freshly prepared Micro BCA reagent was added to the supernatant. The mixture was incubated for 1 h at 60 °C. After cooling to room temperature, the absorbance was measured using an ATI Unicam UV4-100 spectrophotometer (Unicam Instruments Ltd., Cambridge, UK) at 562 nm.

#### 2.4.4. In Vitro Drug Release

The in vitro drug release test was carried out in two types of media, i.e., human blood plasma (pH 7.4) and sodium acetate buffer (pH 5.5). The latter one mimics the microenvironment of the acidic cancer tissues. From the original washed suspension, 0.5 mL was mixed with 4.5 mL of blood plasma and sodium acetate buffer solution in 5 mL non-transparent Eppendorf tubes. 50 µL of 0.3% (*w/v*) sodium azide was added to each sample as bactericide. The samples were incubated for 3 days at 37 °C and shaken at 700 rpm in a Hettich Benelux MKR-13 Thermomixer (Hettich Benelux Laboratory Equipment, Geldermalsen, the Netherlands). Three parallel samples were run.

Aliquots were taken time-to-time (0.25, 0.5, 1, 2, 4, 8, 12, 24, 48, and 72 h), centrifuged and washed three times with MilliQ water. The pellet was dissolved in 1 mL of DMSO and the sorafenib concentration was measured spectrophotometrically. The amount of released cisplatin was measured by ICP-OES, after digesting 0.4 mL of supernatant, as described in Section 2.4.2.

#### 2.4.5. In Vitro Cytotoxicity Study

The cytotoxicity tests in HepG2 cells were performed using the MTT assay. The cells were grown in DMEM medium at 37 °C in a 5% CO_2_ atmosphere. Ninety-six-well plates were used to culture the cells (50,000 cells/well). After 24 h of preincubation, fresh DMEM medium was added; then, after another 1-day incubation, the medium was replaced with fresh DMEM containing the blank or drug-loaded nanoparticles.

Four different drug concentrations (2.5 µg/mL, 5.0 µg/mL, 12.5 µg/mL, and 25.0 µg/mL) were used, while for control samples, the same amount of free CIS and SOR was added. After incubation, 10 µL of MTT solution was added to each well, followed by another incubation for 2 h. The supernatant was removed, and the cells were lysed by MTT lysis solution (DMSO, acetic acid, SDS). The formazan crystals formed in the cells were dissolved, and the absorbance was measured at 570 nm using a Thermo Scientific Multiscan Sky plate reader (Bio-science Kft., Budapest, Hungary) plate reader.

The cell viability was calculated by relating the absorbance of treated cells to the absorbance of untreated cells (negative control). The blank nanoparticle suspension and the pure drug solutions served as positive controls. The data were presented as mean and standard deviation with eight replicates.

#### 2.4.6. Data Processing

The data were classified into four groups, according to the different drug concentrations (2.5 µg/mL, 5.0 µg/mL, 12.5 µg/mL, and 25.0 µg/mL). For the analysis of variances (one-way ANOVA) the normality distribution of variables by the Shapiro–Wilk test and the homogeneity of variances by the Bartlett test was tested. In some cases, when the distribution of data was not normal, the non-parametric Kruskal–Wallis test was used to compare the mean viability between the three different drug containing systems. When the one-way ANOVA or Kruskal–Wallis test showed significant differences in the means, the proper post-hoc test (Tukey-HSD or Kruskal–Nemenyi) was used to identify the actual differences between the pairs. All the statistics were carried out by using R software, version 3.4.0.

## 3. Results and Discussion

### 3.1. Encapsulation of the Active Agents using BSA as Additive

The microencapsulation of BSA in polymeric nanoparticles has been investigated for the assessment of the conditions of the double-emulsion solvent evaporation method. As previously demonstrated by several groups (see the introduction part), specific proteins can be utilised as additives to improve the encapsulation of drugs in polymeric matrices and their delivery. The optimisation of the method was carried out by varying the main preparation parameters, i.e., the ratio of oil and water phase, and encapsulating the polymer concentration, PVA concentration, and BSA amount. A total of 0.5% PVA was found to be insufficient for a suitable size (“12CLBSA1”), hence, 1% emulsifier was applied in the following experiments (Table 1). It was assumed that the oil-in-water ratio plays an important role in the particle size, although, nanoparticles with low size could be produced between 1/3 and 1/5 values. An oil-in-water ratio of 1:3 was appropriate considering the size of nanoparticles (samples “12CLBSA2” and “12CLBSA4”); however, the polydispersity became high, which indicated that other parameters exerted much more crucial effect on size. Too high polymer concentration (2%) resulted in too viscous organic phase and consequently large, aggregated particles (“12CLBSA3”). BSA concentration had substantial influence in the presence of the EDC cross-linker on particle size, probably because the higher concentration of BSA must have resulted in a higher amount adsorbed on the particle surface. The higher number of active groups on the surface enabled enhanced cross-linking, and thus the aggregation of the nanoparticles. Consequently, the encapsulation of 0.5 mg BSA in 10 mg 12CL polymer using 1:5 oil-in-water ratio and 1% PVA emulsifier was considered as the optimal composition for the preparation of nanoparticles with appropriate size and polydispersity (“12CLBSA6”).

Utilisation of the cross-linking agent EDC together with NHS for stabilisation in aqueous solutions was found to be a possible way for improving the coupling of BSA in the polymer matrix by the activation of the polymer. EDC can react with the carboxyl end group of the polymer, forming a reactive O-acylisourea intermediate that reacts further with an amino group of BSA to form an amide group. Unfortunately, the PDI values increased when EDC was added (Table 1, sample “12CLBSA10”). However, this approach was considered reliable to be investigated in the forthcoming experiments, which targeted the encapsulation of the active agents sorafenib (SOR) and cisplatin (CIS), using a similar double emulsion process.

The influence of cross-linking with EDC on the encapsulation efficiency of sorafenib, cisplatin, or both active agents was investigated in the presence of BSA. The size distribution of the obtained samples was mostly wide (Table 2), excepting two samples (“12CLBSASORCIS2” and “12CLBSACIS3”), where a particle size of about 200 nm and a very narrow size distribution were reached. However, in these samples the yield of forming polymer suspension was too low (data not shown). The coupling with EDC increased the water solubility of nanoparticles; hence, the particle yield became very low.

**Figure 1:**
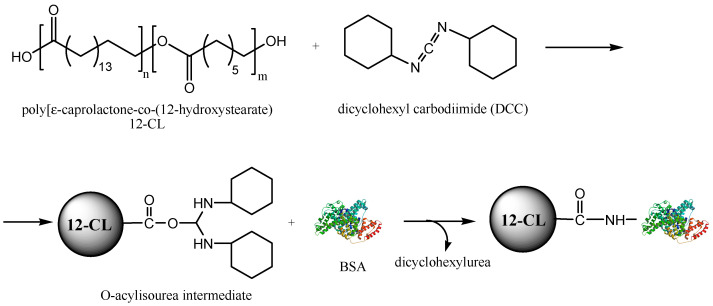
Reaction scheme of the 12CL polymer activation with BSA by cross-linking with dicyclohexyl carbodiimide (DCC).

To prevent the increase of water solubility of the cross-linked polymer particles by EDC, in the following experiments the utilisation of a water-insoluble cross-linker, DCC was investigated. The samples were prepared using the same process conditions as with EDC. The mechanism of DCC cross-linking is similar to that of EDC, involving the formation of an O-acylisourea intermediate that further reacts with a primary amine group of the BSA protein, forming a covalent amide bond (Figure 1). BSA can be replaced as drug encapsulation additive by another protein or peptide. It must also be specified that the attachment of BSA to the polymer is completed by the formation of hydrogen bonds and hydrophobic interactions.

**Figure 2:**
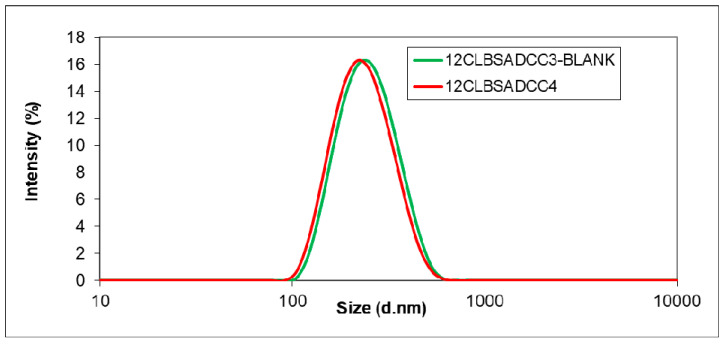
Particle size distribution of BSA encapsulated nanoparticles using DCC as cross-linker (red line) and the blank sample (green line).

Replacing EDC by DCC, the water solubility of the particles was avoided, and the encapsulation efficiency (EE) increased up to 82% when 2 mg DCC were used (Table 3). The results show that the use of DCC was beneficial for BSA microencapsulation, and the process provided nanoparticles with small size and monodisperse distribution (Figure 2) accompanied by high encapsulation efficiency.

Further, the simultaneous encapsulation of both active agents together with BSA was studied under the same conditions as the previously described blank samples using DCC as a cross-linker. As shown in Table 4, the utilisation of DCC (sample “12CLBSACISH5”) increased the production yield and slightly increased the encapsulation efficiency compared to the nanoparticles produced without DCC (sample “12CLBSACISH4”). It can be concluded that the encapsulation of both active agents was successfully accomplished using 12CL carrier and BSA, in which the protein was applied to multiply the binding sites in the nanoparticles.

### 3.2. Simultaneous Encapsulation of Sorafenib and Cisplatin using iRGD

After collecting sufficient information on the double emulsion process using BSA protein, SOR and CIS were co-encapsulated by the 12CL encapsulating polymer, and BSA was replaced by the iRGD tumour homing peptide. A suitable size distribution and polydispersity index values were obtained (Table 5). The higher iRGD amount (sample “DCCRGD2”) gave higher nanoparticle yield (59%), while the particle size and polydispersity of nanoparticles did not change significantly. We hypothesize that the higher amount of encapsulated iRGD provided higher concentration of active groups that could be cross-linked by the encapsulating polymer. Hence, the dissolution of the more water-soluble portion of the 12CL copolymer (oligomers with lower molecular weight) was partly inhibited via the cross-linking with iRGD.

Before the microencapsulation process, cisplatin was hydrolysed by mixing it with the iRGD solution, and stirred for one night to promote the binding. The reason for this experimental approach was that a bigger conjugated molecule can probably be encapsulated more efficiently with the double emulsion solvent evaporation method than a single compound; moreover, the encapsulation efficiency could be increased, and the iRGD peptide can also serve as targeting agent during the drug delivery. The hydrolysis of CIS could also improve the anticancer efficiency, as it is considered to play a key role in its anticancer activity [29].

**Figure 3:**
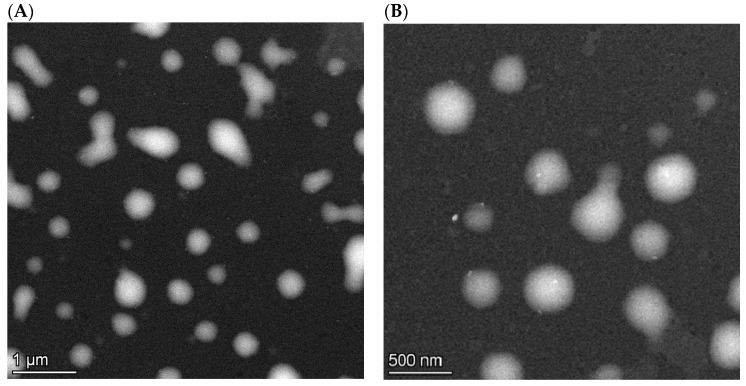
S/TEM images of iRGD, sorafenib, and cisplatin-loaded nanospheres (scale bars: (**A**): 1 μm, (**B**): 500 nm).

Microencapsulating the active agents with iRGD resulted in nanospheres (Figure 3) with appropriate particle size and polydispersity (Table 6). Unfortunately, the imaging of the nanoparticles was not an easy task probably due to the charging of the polymeric carrier; hence, the S/TEM images were blurry (Figure 3), and some particles became deliquescent under the electron beam. Therefore, the hydrodynamic size data provided by DLS have been evaluated as more reliable, which showed Z-avg values between 206 nm and 221 nm. Babu et al. [30] co-entrapped paclitaxel and cisplatin by the PLGA-chitosan carrier with the emulsion solvent evaporation method, using iRGD as additive, and obtained a similar average particle size, close to 200 nm.

**Figure 4:**
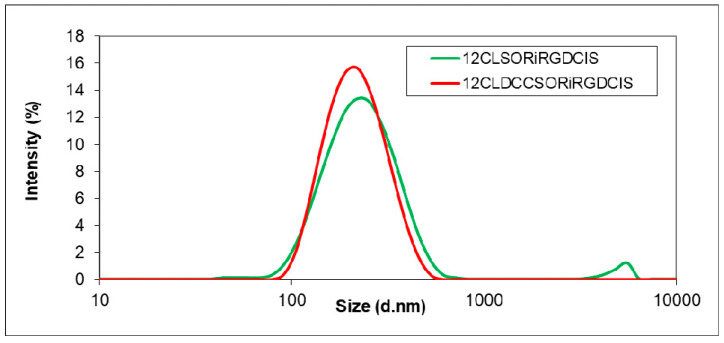
Particle size distribution of dual drug and iRGD-loaded nanoparticles with (DCCSORiRGDCIS) and without DCC (SORiRGDCIS).

The encapsulation efficiency was found to be 54–55% and 23–25% for sorafenib and cisplatin, respectively. The drug loading capacity for cisplatin was 1.6 ± 0.3% for the sample “SORiRGDCIS”. In this case, the utilisation of the DCC cross-linker (sample “DCCSORiRGDCIS”) did not improve the encapsulation efficiency of the active agents, which remained practically unchanged. Surprisingly, the encapsulation efficiency of the iRGD peptide was substantially decreased in the presence of DCC (Table 6). As mentioned before, the cross-linking with DCC involves the formation of amide bonds, although the attachment of iRGD to the polymer can also occur by hydrogen bonding and hydrophobic interactions between nonpolar groups. Apparently, in the case of iRGD, a 9-amino acid cyclic peptide, the covalent bonding results in a lower amount of attached peptide, also hindering the other coupling possibilities. At the same time, the average size of the particles prepared with DCC decreased slightly (Figure 4) and the size distribution was improved, as shown by the PDI values as well (Table 6). The co-entrapment of active agents did not change the zeta potential of nanoparticles. As the encapsulation of the active agents (not of iRGD) was the main target, these results indicate that both investigated variants can be used with the same efficiency, allowing the selection in accordance with the specific requirements of the process.

According to the available literature data, Tian et al. [31] co-encapsulated the paclitaxel and cisplatin in PLGA, using the nanoprecipitation method. The drug loading was 1.9 ± 0.1%, 1.7 ± 0.1% for pure cisplatin and the co-encapsulated system, respectively. Callari et al. [32] conjugated cisplatin to PLGA-mPEG nanoparticles with a drug loading capacity of 0.5–1%. Moreno et al. [33] and Jayasuriya and Darr [34] reached an encapsulation efficiency of 11.2% and 30%, respectively, for cisplatin, both using the double emulsion method. Compared to the above-mentioned reports, the results of our present study reaching a 1.6% drug loading capacity and 25% encapsulation efficiency for cisplatin are at the similar levels. It must be also pointed out that co-encapsulation of sorafenib and cisplatin in bioconjugated systems has not yet been reported.

### 3.3. Drug Release Study

**Figure 5:**
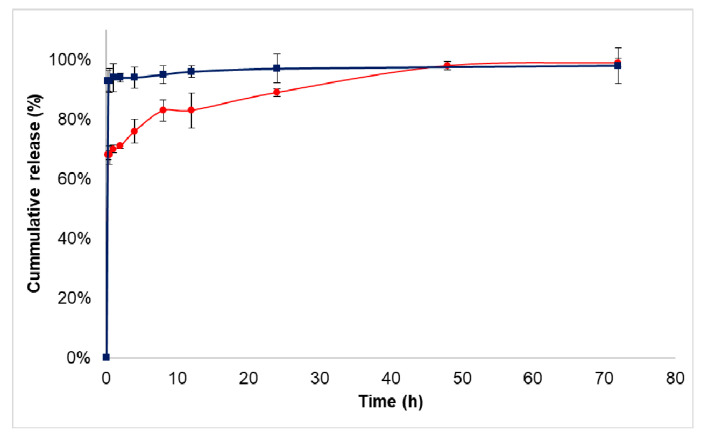
Cumulative drug release of sorafenib from dual drug-loaded 12CL nanoparticles in sodium acetate buffer at pH 5.5 (red) and in blood plasma at pH 7.4 (blue).

The in vitro drug release of co-encapsulated nanoparticles was investigated under biorelevant condition in blood plasma and acidic sodium acetate buffer mimicking the tumour microenvironment. For the release study, the bioconjugate showing the best encapsulation properties (sample “SORiRGDCIS”) was used. The initial burst of sorafenib in sodium acetate buffer solution was 68 ± 1.5%; then, a sustained release of the active agent occurred due to the polymer degradation and the diffusion. During the 72-h study, 99 ± 1.5% of the total drug was released (Figure 5). In blood plasma, 93 ± 3.4% of the drug was released in the initial burst phase.

In the case of cisplatin, more than 90% of the active agent was released in the initial burst phase in both media (data not shown). Avgoustakis et al. [35] and Moreno et al. [33] described the same cisplatin release profile with a rapid initial burst. After the cellular uptake of the cisplatin, it gains activity during aquation. The chloride ligands are substituted with water molecules; then, they bind to the cytosine-guanine (C-G) DNA base pair. Cisplatin preferentially binds to guanine [36]. The high burst effects can be explained with a strong affinity of the active agents to the plasma proteins; moreover, most of the cisplatin is probably adsorbed on the surface of nanoparticles. The microencapsulation of cisplatin, a highly water-soluble substance that uses polymeric nanoparticles, also provided a great challenge for other research groups [33,35]. To co-encapsulate another anticancer agent enlarges the difficulties, although it can add substantial synergistic value to the nanoparticulate system. Sorafenib as a poorly water-soluble drug has got a low bioavailability, which has been significantly improved by the entrapment into 12CL copolymer, which was proved by the fast release of drugs. However, the experienced especially high initial burst can be harmful if the drugs are released far away from the target site. Hence, with such a nanosystem an administration route done close to the target organ is suggested, which must help avoid serious side effects. Additionally, the involvement of a tumour penetrating iRGD peptide in the system might contribute to the efficient targeting of the nanoparticles to the tumour cells.

### 3.4. Cytotoxicity Test

**Figure 6:**
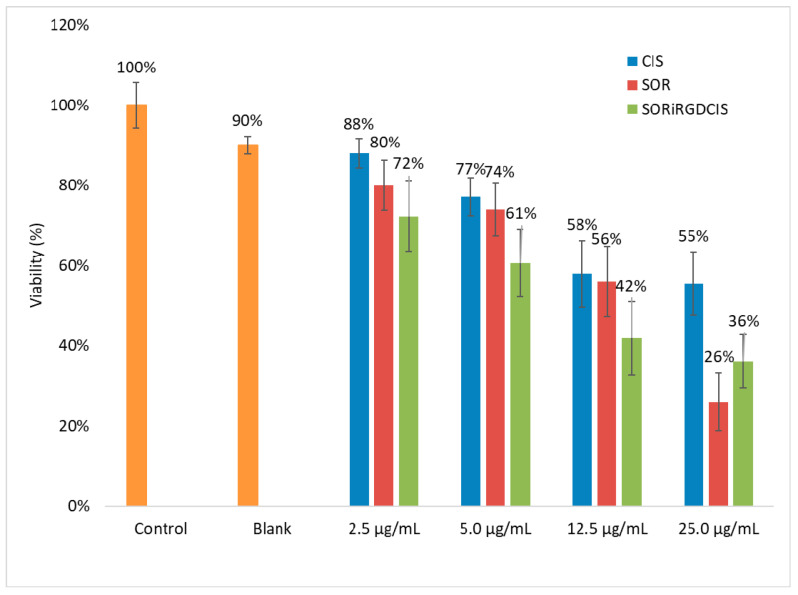
Cytotoxicity of dual drug-loaded 12CL nanoparticles (SORiRGDCIS) and the free drugs (CIS-cisplatin, SOR-sorafenib) studied in HepG2 cells.

The in vitro cytotoxicity test was carried out using HepG2 cells to investigate the efficiency of the dual drug-loaded nanoparticles. Untreated cells were used as a negative control, while the 12CL blank sample and the pure drug solution served as positive controls (Figure 6). The nanoparticles showed concentration-dependent cytotoxicity. The dual drug system was more efficient at most concentration level than the pure drug, which can be seen from the viability values. With the highest drug concentration, the cell viability of CIS was 55 ± 8%, while at the co-entrapped CIS-SOR, the viability decreased significantly to 36 ± 7%. The statistical comparison of the mean viability between the three drug systems in each concentration groups (Table 7) demonstrated a significantly higher viability for the single drug cisplatin compared with the dual drug system. Significant difference was between the sorafenib and dual drug system in the 5.0 µg/mL and 12.5 µg/mL groups. In the highest concentration cisplatin showed a significantly higher viability than sorafenib.

It must be noted that at the highest sorafenib concentration, the viable cells were reduced more effectively, probably because at this concentration level the drug release of nanoparticles also influenced the toxicity, as the pure drug is available immediately for the cells, while the nanoparticles can delay the drug availability. Nevertheless, in a living system, the use of nanoparticles can be advantageous, because they do not release the total drug to the healthy tissues, and in the case of an effective targeting they can transport most of the chemotherapeutics to the tumour cells.

## 4. Conclusions

A double emulsion–solvent evaporation method for the preparation of sorafenib- and cisplatin-loaded 12CL copolymer nanoparticles was developed. The model compound BSA was microencapsulated and cross-linked using DCC by the copolymer. Under similar conditions, cisplatin and sorafenib were successfully co-encapsulated by the double emulsion method using 12CL, while an iRGD tumour penetrating peptide was also incorporated instead of BSA. The presence of active agents led to the decrease of particle size. The use of DCC improved the encapsulation efficiency and system stability. Small particle size and narrow size distribution were obtained, and high encapsulation efficiency was performed for both of the anticancer drugs and the tumour homing peptide. In the in vitro drug release study, a higher initial burst could be observed due to the high affinity to plasma proteins. The dual drug-loaded nanoparticles showed concentration-dependent cytotoxicity and were similarly efficient to the pure chemotherapeutic drug. The next step of the present development is to investigate the performance of iRGD-functionalised and dual drug-loaded nanoparticles in in vivo studies.

## Figures and Tables

**Table 1:** Optimisation of the nanoparticle preparation method by BSA encapsulation.

Sample	o/w Ratio	Polymer [%]	PVA [%]	BSA [mg]	EDC [mg]	Z-avg [nm]	PDI
12CLBSA1	1/2	1	0.5	1	0	476.6 ± 9.3	0.450 ± 0.018
12CLBSA2	1/3	1	1	1	0	241.3 ± 5.8	0.341 ± 0.004
12CLBSA3	1/3	2	1	1	0	481.9 ± 39.6	0.692 ± 0.022
12CLBSA4	1/3	1	1	0.5	0	232.3 ± 10.2	0.360 ± 0.051
12CLBSA5	1/5	1	1	1	0	269.9 ± 7.8	0.355 ± 0.019
12CLBSA6	1/5	1	1	0.5	0	245.5 ± 3.1	0.275 ± 0.007
12CLBSA7	1/4	1	1	1	0	301.2 ± 7.6	0.461 ± 0.018
12CLBSA8	1/4	1	1	0.5	0	338.8 ± 11.6	0.568 ± 0.035
12CLBSA9	1/5	1	1	1	1	438.5 ± 25.1	0.810 ± 0.121
12CLBSA10	1/5	1	1	0.5	1	250.7 ± 8.3	0.353 ± 0.038
12CLBlank	1/5	1	1	0	0	215.2 ± 1.4	0.222 ± 0.011

**Table 2:** Encapsulation of the active agents, using 1:5 oil-in-water ratio, 1% PVA emulsifier, and 0.5 mg BSA.

Sample	Sorafenib [mg]	Cisplatin [mg]	EDC [mg]	Z-avg [nm]	PDI
12CLBSASOR1	1	0	0	223.4 ± 2.4	0.243 ± 0.008
12CLBSASOR2 ^a^	1	0	1	274.4 ± 8.6	0.582 ± 0.018
12CLBSASOR3 ^b^	1	0	1	212.3 ± 0.8	0.258 ± 0.028
12CLBSASORCIS1	0.5	0.5	0	239.6 ± 3.7	0.378 ± 0.014
12CLBSASORCIS2 ^a^	0.5	0.5	1	196.1 ± 1.7	0.081 ± 0.013
12CLBSASORCIS3 ^b^	0.5	0.5	1	249.3 ± 8.1	0.362 ± 0.021
12CLBSACIS1	0	1	0	252.2 ± 15.0	0.413 ± 0.02
12CLBSACIS2 ^a^	0	1	1	246.3 ± 27.2	0.397 ± 0.070
12CLBSACIS3 ^b^	0	1	1	188.9 ± 2.0	0.091 ± 0.008

^a^ EDC added to the inner water phase. ^b^ EDC added to the outer water phase.

**Table 3:** Encapsulation of BSA by polymer activation with DCC cross-linker.

Sample	BSA [mg]	DCC [mg]	Z-avg [nm]	PDI	EE BSA [%]	Yield [%]
12CLBSADCC1	0.5	1	217.6 ± 2.0	0.129 ± 0.014	71	-
12CLBSADCC2	0.5	2	225.9 ± 2.2	0.115 ± 0.015	73	46
12CLBSADCC3	1	2	219.7 ± 1.7	0.121 ± 0.011	82	46
12CLBSADCCBlank	0	2	233.4 ± 1.4	0.136 ± 0.020	-	47

**Table 4:** Simultaneous encapsulation of the active agents sorafenib and cisplatin with BSA additive and DCC as cross-linker, using 1:5 oil-in-water ratio, 1% (*w/v*) 12CL encapsulating polymer concentration, and 1% (*w/v*) PVA emulsifier.

Sample	Cisplatin [mg]	DCC [mg]	Z-avg [nm]	PDI	Yield [%]	EE Cisplatin [%]
12CLBSACISH4	0.5	-	204.6 ± 1.1	0.250 ± 0.014	40	24
12CLBSACISH5 Blank	-	2	212.9 ± 2.4	0.171 ± 0.019	39	-
12CLBSACISH5	0.5	2	209.8 ± 3.0	0.209 ± 0.015	61	28

**Table 5:** Encapsulation of the iRGD peptide with DCC using 1:5 oil-in-water ratio, 1% (*w/v*) 12CL polymer concentration, and 1% (*w/v*) PVA emulsifier.

Sample	iRGD [mg]	Z-avg [nm]	PDI	Zeta Potential [mV]	Yield [%]
DCCRGD Blank	-	210.8 ± 1.6	0.119 ± 0.019	−11.00	59
DCCRGD1	0.5	223.7 ± 3.0	0.143 ± 0.03	−11.20	49
DCCRGD2	1	218.1 ± 0.9	0.158 ± 0.014	−11.80	59

**Table 6:** Encapsulation of active agents and iRGD tumour homing peptide by 12CL carrier.

Sample	Z-avg [nm]	PDI	Zeta Potential [mV]	Yield [%]	EE Sorafenib [%]	EE Cisplatin [%]	EE iRGD [%]
iRGDBlank	220.6 ± 4.2	0.256 ± 0.016	−9.42	63 ± 12	-	-	-
SORiRGDCIS	220.8 ± 2.5	0.221 ± 0.021	−10.0	65 ± 11	54 ± 1.0	25 ± 1.0	42 ± 3.0
DCCSOR-iRGDCIS	205.9 ± 2.8	0.148 ± 0.012	−11.8	74 ±13	55 ± 2.8	23 ± 2.1	29 ± 3.1

**Table 7:** One-way-ANOVA/Kruskal–Wallis test performed for each drug concentration group (C—cisplatin, S—sorafenib, and CS—cisplatin-sorafenib dual drug system).

Concentration Group	Normality Test (Shapiro–Wilk)	Homoscedasticity (Bartlett Test)	One-way-ANOVA/Kruskal–Wallis	Post-hoc (Tukey HSD/Nemenyi or Dunn)
2.5 µg/mL	C: *p* = 0.440 S: *p* = 0.630 CS: *p* = 0.694	*p* = 0.219	F = 7.922, df = 2, *p* < 0.01 **	C-CS: *p* < 0.01 **
5.0 µg/mL	C: *p* = 0.296 S: *p* = 0.374 CS: *p* = 0.027	*p* = 0.158	Chi-sq = 13.03, df = 2, *p* < 0.01 **	C-CS: *p* < 0.01 ** S-CS: *p* < 0.05 *
12.5 µg/mL	C: *p* = 0.041 S: *p* = 0.307 CS: *p* = 0.350	*p* = 0.466	Chi-sq = 14.25, df = 2, *p* < 0.001 ***	C-CS: *p* < 0.01 ** S-CS: *p* < 0.01 **
25.0 µg/mL	C: *p* = 0.048 S: *p* = 0.227 CS: *p* = 0.829	*p* = 0.321	Chi-sq = 18.12, df = 2, *p* < 0.001 ***	C-S: *p* < 0.05 * C-CS: *p* < 0.001 ***

*, *p* < 0.05; **, *p* < 0.01, and ***, *p* < 0.001.

## Data Availability

The data presented in this study are available on request from the corresponding authors.

## Supplementary Materials

The following are available online at https://www.mdpi.com/article/10.3390/biomedicines10010043/s1, Figure S1: MALDI-TOF MS spectrum of the 12CL copolymer used for the nanoencapsulation of sorafenib and cisplatin. In the excerpt, the 1000–3000 m/z domain, indicating the linear copolymers with one (triangles) and two (circles) 12-hydroxystearic unit in the backbone.

## References

[1] Wicki A., Witzigmann D., Balasubramanian V., Huwyler J. (2015). Nanomedicine in cancer therapy: Challenges, opportunities, and clinical applications. J. Control Release.

[2] Sinha R., Kim G.J., Nie S., Shin D.M. (2006). Nanotechnology in cancer therapeutics: Bioconjugated nanoparticles for drug delivery. Mol. Cancer Ther..

[3] Esfahani D.R., Tangen K.M., Sadeh M., Seksenyan A., Neisewander B.L., Mehta A.I., Linninger A.A., Manca D. (2018). Systems engineers’ role in biomedical research. Convection-enhanced drug delivery. Computer Aided Chemical Engineering.

[4] Seo S.-J., Chen M., Wang H., Kang M.S., Leong K.W., Kim H.-W. (2017). Extra- and intra-cellular fate of nanocarriers under dynamic interactions with biology. Nano Today.

[5] Feczkó T., Piiper A., Pleli T., Schmithals C., Denk D., Hehlgans S., Rödel F., Vogl T.J., Wacker M.G. (2019). Theranostic Sorafenib-Loaded Polymeric Nanocarriers Manufactured by Enhanced Gadolinium Conjugation Techniques. Pharmaceutics.

[6] Moghimi S.M., Peer D., Langer R. (2011). Reshaping the future of nanopharmaceuticals: Ad Iudicium. ACS Nano.

[7] Cole J.T., Holland N.B. (2015). Multifunctional nanoparticles for use in theranostic applications. Drug Deliv. Transl. Res..

[8] Tang S., Li Y. (2019). Sorafenib-Loaded Ligand-Functionalized Polymer-Lipid Hybrid Nanoparticles for Enhanced Therapeutic Effect Against Liver Cancer. J. Nanosci. Nanotechnol..

[9] Forner A., Gilabert M., Bruix J., Raoul J.-L. (2014). Treatment of intermediate-stage hepatocellular carcinoma. Nat. Rev. Clin. Oncol..

[10] Gbolahan O.B., Schacht M.A., Beckley E.W., LaRoche T.P., O’Neil B.H., Pyko M. (2017). Locoregional and systemic therapy for hepatocellular carcinoma. J. Gastrointest. Oncol..

[11] Keating G.M., Santoro A. (2009). Sorafenib: A Review of its Use in Advanced Hepatocellular Carcinoma. Drugs.

[12] Duan X., He C., Kron S.J., Lin W. (2016). Nanoparticle formulations of cisplatin for cancer therapy. Wiley Interdiscip. Rev. Nanomed. Nanobiotechnol..

[13] Alam N., Khare V., Dubey R., Saneja A., Kushwaha M., Singh G., Sherma N., Chandan B., Gupta P.N. (2014). Biodegradable polymeric system for cisplatin delivery: Development, in vitro characterization and investigation of toxicity profile. Mater. Sci. Eng. C Mater. Biol. Appl..

[14] Gryparis E.C., Mattheolabakis G., Bikiaris D., Avgoustakis K. (2007). Effect of Conditions of Preparation on the Size and Encapsulation Properties of PLGA-mPEG Nanoparticles of Cisplatin. Drug Deliv..

[15] Senapati S., Mahanta A.K., Kumar S., Maiti P. (2018). Controlled drug delivery vehicles for cancer treatment and their performance. Sig. Transduct. Target. Ther..

[16] Attia M.F., Anton N., Wallyn J., Omran Z., Vandamme T.F. (2019). An overview of active and passive targeting strategies to improve the nanocarriers efficiency to tumour sites. J. Pharm. Pharmacol..

[17] Kaur I.P., Singh H. (2014). Nanostructured drug delivery for better management of tuberculosis. J. Control Release.

[18] ud Din F., Aman W., Ullah I., Qureshi O.S., Mustapha O., Shafique S., Zeb A. (2017). Effective use of nanocarriers as drug delivery systems for the treatment of selected tumors. Int. J. Nanomed..

[19] Martinez N.Y., Andrade P.F., Durán N., Cavalitto S. (2017). Development of Double Emulsion Nanoparticles for the Encapsulation of Bovine Serum Albumin. Colloids Surf. B.

[20] Wang Y., Chen S., Yang X., Zhang S., Cui C. (2021). Preparation optimization of bovine serum albumin nanoparticles and its application for siRNA delivery. Drug Des. Dev. Ther..

[21] Byrne J.D., Betancourt T., Brannon-Peppas L. (2008). Active targeting schemes for nanoparticle system in cancer therapeutics. Adv. Drug Deliv. Rev..

[22] Gao F., Zhang J., Fu C., Xie X., Peng F., You J., Tang H., Wang Z., Li P., Chen J. (2017). iRGD-modified lipid-polymer hybrid nanoparticles loaded with isoliquiritigenin to enhance anti-breast cancer effect and tumor-targeting ability. Int. J. Nanomed..

[23] Zhong Y., Su T., Shi Q., Feng Y., Tao Z., Huang Q., Li L., Hu L., Li S., Tan H. (2019). Co-Administration Of iRGD Enhances Tumor-Targeted Delivery and Anti-Tumor Effects of Paclitaxel-Loaded PLGA Nanoparticles for Colorectal Cancer Treatment. Int. J. Nanomed..

[24] Todea A., Aparaschivei D., Badea V., Boeriu C.G., Peter F. (2018). Biocatalytic route for the synthesis of oligoesters of hydroxy-fatty acids and ϵ-caprolactone. Biotechnol. J..

[25] Kántor I., Aparaschivei D., Todea A., Biró E., Babos G., Szerényi D., Kakasi B., Péter F., Șișu E., Feczkó T. (2021). Biocatalytic synthesis of poly[ε-caprolactone-co-(12-hydroxystearate)] copolymer for sorafenib nanoformulation useful in drug delivery. Catal. Today.

[26] Todea A., Dreavă D.M., Benea I.C., Bîtcan I., Peter F., Boeriu C.G. (2021). Achievements and trends in biocatalytic synthesis of specialty polymers from biomass-derived monomers using lipases. Processes.

[27] Hevilla V., Sonseca A., Echeverría C., Muñoz-Bonilla A., Fernández-García M. (2021). Enzymatic synthesis of polyesters and their bioapplications: Recent advances and perspectives. Macromol. Biosci..

[28] Todea A., Otten L.G., Frissen A.E., Arends I.W.C.E., Peter F., Boeriu C.G. (2015). Selectivity of lipases for estolides synthesis. Pure Appl. Chem..

[29] Du Y., Zhang N., Cui M., Liu Z., Liu S. (2012). Investigation on the hydrolysis of the anticancer drug cisplatin by Fourier transform ion cyclotron resonance mass spectrometry. Rapid Commun. Mass Spectrom..

[30] Babu A., Amreddy N., Muralidharan R., Pathuri G., Gali H., Chen A., Zhao Y.D., Munshi A., Ramesh R. (2017). Chemodrug delivery using integrin-targeted PLGA-Chitosan nanoparticle for lung cancer therapy. Sci. Rep..

[31] Tian J., Min Y., Rodgers Z., Man Au K., Hagan C.T., Zhang M., Roche K., Yang F., Wagner K., Wang A.Z. (2017). Co-delivery of paclitaxel and cisplatin with biocompatible PLGA-PEG nanoparticles enhances chemoradiotherapy in non-small cell lung cancer models. J. Mater. Chem. B.

[32] Callari M., Aldrich-Wright J.R., de Souza P.L., Stenzel M.H. (2014). Polymers with platinum drugs and other macromolecular metal complexes for cancer treatment. Prog. Polym. Sci..

[33] Moreno D., Tros de Ilarduya C., Bandrés A., Buñuales M., Azcona M., García-Foncillas J., Garrido M.J. (2008). Characterization of cisplatin cytotoxicity delivered from PLGA-system. Eur. J. Pharm. Biopharm..

[34] Jayasuriya A., Darr A. (2013). Controlled release of cisplatin and cancer cell apoptosis with cisplatin encapsulated poly(lactic-co-glycolic acid) nanoparticles. J. Biomed. Sci. Eng..

[35] Avgoustakis K., Beletsi A., Panagi Z., Klepetsanis P., Karydas A.G., Ithakissios D.S. (2002). PLGA-mPEG nanoparticles of cisplatin: In vitro nanoparticle degradation, in vitro drug release and in vivo drug residence in blood properties. J. Control Release.

[36] Johnstone T.C., Suntharalingam K., Lippard S.J. (2016). The Next Generation of Platinum Drugs: Targeted Pt(II) Agents, Nanoparticle Delivery, and Pt(IV) Prodrugs. Chem. Rev..

