# Supplementary material for: Co-Entrapment of Sorafenib and Cisplatin Drugs and iRGD Tumour Homing Peptide by Poly[ε-caprolactone-co-(12-hydroxystearate)] Copolymer
Source: Biomedicines. 2021 Dec 26;10(1):43. doi: 10.3390/biomedicines10010043 (PMC8772891; doi:10.3390/biomedicines10010043)
Supplement: Supplementary file 1 [file biomedicines-10-00043-s001.zip › biomedicines-1491487-supplementary.pdf]

# Co-entrapment of sorafenib and cisplatin drugs and iRGD tumour homing peptide by poly[ε-caprolactone-co-(12-hydroxystearate)] copolymer


**Izolda Kántor[1,2], Diana Dreavă[3], Anamaria Todea[3], Francisc Péter[3,4*], Zoltán May[1], Emese Biró[3], György Babos[2], Tivadar Feczkó[1,2*]**

[1]Institute of Materials and Environmental Chemistry, Research Centre for Natural Sciences, Magyar tudósok körútja 2, H-1117, Budapest, Hungary.

[2]Research Institute of Biomolecular and Chemical Engineering, University of Pannonia, Egyetem u. 10, H-8200, Veszprém, Hungary.

[3]University Politehnica of Timişoara, Faculty of Industrial Chemistry and Environmental Engineering, Carol Telbisz 6, RO-300001, Timişoara, Romania.

[4]Research Institute for Renewable Energies, University Politehnica Timișoara, G. Muzicescu 138, Timișoara, RO-300501, Romania;

* Correspondence: anamaria.todea@upt.ro, feczko@mukki.richem.hu


## Supplementary material

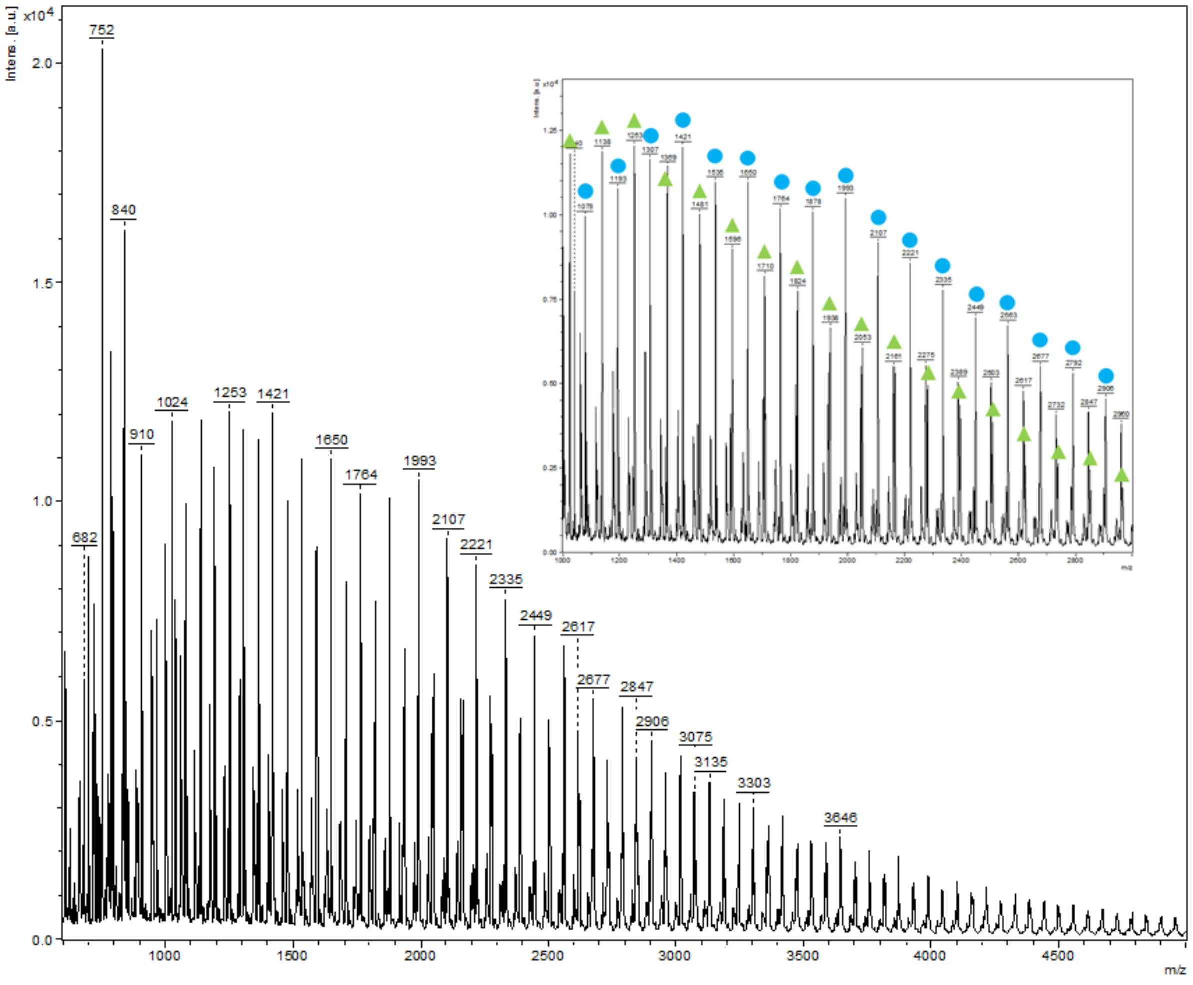


**Figure S1.** MALDI-TOF MS spectrum of the 12CL copolymer used for nanoencapsulation of sorafenib and cisplatin. In the excerpt, the 1000-3000 m/z domain, indicating the linear copolymers with one (triangles) and two (circles) 12-hydroxystearic unit in the backbone.
